# Height Measurement Method for Meter-Wave Multiple Input Multiple Output Radar Based on Transmitted Signals and Receive Filter Design

**DOI:** 10.3390/s25020478

**Published:** 2025-01-15

**Authors:** Cong Qin, Qin Zhang, Guimei Zheng, Xiaolong Fu, He Zheng

**Affiliations:** 1 Air and Missile Defense College, Air Force Engineering University, Xi’an 710051, China; qc337810137@163.com (C.Q.); kinzh@263.net (Q.Z.); fuxiaolong_12@163.com (X.F.); littlezhengh@163.com (H.Z.); 2 School of Computer Science and Technology, Weinan Normal University, Weinan 714099, China; 3 Graduate College, Air Force Engineering University, Xi’an 710051, China

**Keywords:** height measurement, beamforming technology, cognitive processing method, joint optimization of transmitted signal and receive filter, MIMO radar

## Abstract

To address the issue of low-elevation target height measurement in the Multiple Input Multiple Output (MIMO) radar, this paper proposes a height measurement method for meter-wave MIMO radar based on transmitted signals and receive filter design, integrating beamforming technology and cognitive processing methods. According to the characteristics of beamforming technology forming nulls at interference locations, we assume that the direct wave and reflected wave act as interference signals and hypothesize a direction for a hypothetical target. Then, the data received are processed to obtain the height of low-elevation-angle targets using a cognitive approach that jointly optimizes the transmitted signal and receive filter. Firstly, a signal model for the meter-wave MIMO radar based on the transmit weight matrix is established under low-elevation scenarios. Secondly, the signal model is analyzed and transformed. Thirdly, the beamforming algorithm that jointly optimizes the transmitted signals and receive filter is derived and analyzed. The algorithm maximizes the output Signal-to-Interference-plus-Noise ratio (SINR) of the receiver by designing the transmit weight matrix and receive filter. The optimization problem based on the SINR criterion is non-convex and difficult to solve. We transformed it into two sub-optimization problems and approximated the optimal solution through an alternating iteration algorithm. Finally, the proposed height measurement algorithm is compared with the Generalized Multiple Signal Classification (GMUSIC) and Maximum Likelihood (ML) height measurement algorithms. Simulation results show that the proposed algorithm can realize the height measurement of low-elevation targets. Compared to the GMUSIC and ML algorithms, it demonstrates superior performance in terms of computational complexity and multi-target elevation estimation.

## 1. Introduction

The direction of arrival (DOA) estimation [1,2] is an important branch in array signal processing, widely applied in fields such as radar and sonar. The problem of height measurement based on DOA estimation for low-elevation targets is a critical issue in this field. In the direction of finding and tracking low-elevation targets, multipath effects severely impact the radar’s ability to accurately acquire the true position of the target, leading to a decline in the performance of radar direction finding and tracking. Due to the close angular spacing and coherence between the direct signal and its mirror image, the estimation of elevation angles for low-elevation targets has always been a challenge. Existing direction of arrival (DOA) estimation methods for low-elevation targets can mainly be categorized into three types: subspace-based algorithms [3,4], maximum likelihood methods [5,6], and compressive sensing-based algorithms [7,8]. With the deepening research on MIMO radar in recent years, researchers have gradually discovered the potential of this special radar system to address multipath effects. Unlike the traditional meter-wave radar, the meter-wave MIMO radar needs to consider both a transmit multipath and receive multipath simultaneously [9,10]. This makes some traditional algorithms, such as Multiple Signal Classification (MUSIC) [11], ineffective. To address this, the authors of [12] proposed a GMUSIC algorithm that reconstructs the synthesized steering vectors orthogonal to the noise subspace, even in the presence of coherent sources. The maximum likelihood estimation algorithm [13] can be directly applied to the MIMO radar. However, these algorithms require substantial computational effort. Additionally, a height measurement method for low-angle targets based on compressed sensing is proposed in [14] by combining rank-1 constraint techniques. Furthermore, the authors of [15] studied a practical signal model and proposed a matrix pencil-based height measurement method. It is worth noting that traditional algorithms regard multipath as interference and suppress it. In contrast, time reversal (TR) technology takes a different approach by utilizing multipath effects and finding extensive applications in the low-angle direction of arrival estimation [16,17,18]. Adaptive beamforming technology [19], with its excellent interference resistance capabilities, is relatively mature. In [20], multipath echo signals are treated as interference signals in the application scenario. By utilizing adaptive beamforming technology, the multipath echo signals are canceled to obtain the elevation angle of the direct wave.

**Figure 1:**
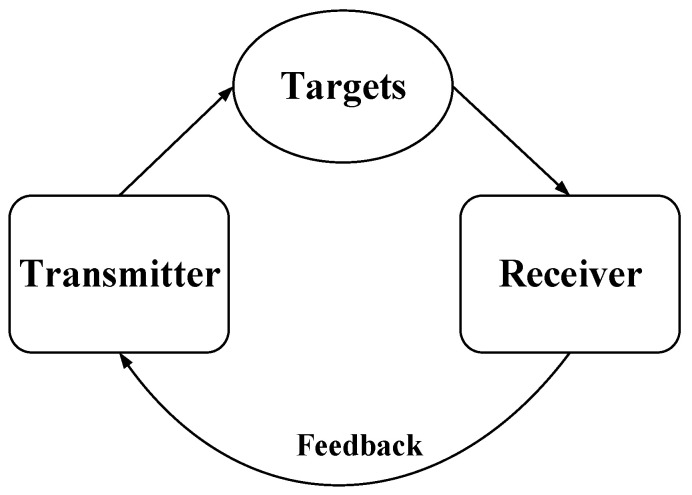
Cognitive loop.

Additionally, compared to traditional radar, a cognitive radar [21] is capable of acquiring information about the surrounding electromagnetic environment and adaptively adjusting certain parameters of the radar to perceive changes in the environment, thereby enhancing radar performance. A cognitive loop is proposed in [22], where the loop begins with the transmitter illuminating the radar environment. Subsequently, the receiving system adaptively probes the actual environment by detecting radar echoes to obtain necessary information about the targets. This information is then fed back from the receiving system to the transmitting system. Finally, the transmitting and receiving systems adjust their parameters according to the feedback information. The cognitive loop is shown in Figure 1. In the cognitive loop, the use of adaptive methods to adjust the radar state to accommodate changing environments forms a key component of the cognitive framework.

**Figure 2:**
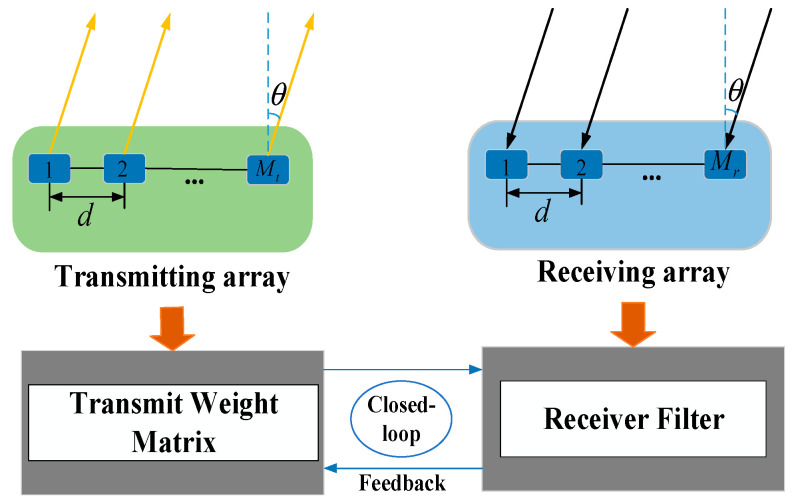
The framework of the beamforming algorithm based on the joint optimization of the transmit weight matrix and receive filter.

Cognitive methods have been extensively studied for the traditional Phased Array (PA) radar and MIMO radar [23,24,25,26,27,28,29,30,31,32,33,34,35,36]. Combining beamforming technology and cognitive processing methods, this paper proposes a height measurement method for meter-wave MIMO radar based on transmitted signals and receive filter design. According to the characteristics of beamforming technology forming nulls at interference locations, we assume that the direct wave and the reflected wave act as interference signals and hypothesize a direction for the target. Then, using a cognitive approach that jointly optimizes the transmitted signal and receive filter, we processed the received data to obtain the height of a low-elevation-angle target. The framework of the beamforming algorithm based on the joint optimization of the transmitted signal and receive filter is shown in Figure 2. It is important to clarify that, in order to better illustrate the joint optimization of the beamforming algorithm of the transmit weight matrix and the receive filter, we depicted the transmitting and receiving antennas separately despite their co-located physical arrangement.

This paper is organized as follows. First, a signal model for the meter-wave MIMO radar, based on the transmit weight matrix, is established for low-elevation scenarios. Second, the signal model is analyzed and transformed. Third, the beamforming algorithm for the joint optimization of the transmitted signal and receive filter in MIMO radar is derived and analyzed. We aimed to maximize the SINR of the receiver by designing the transmit weight matrix and the receive filter. The optimization problem based on the SINR criterion is non-convex and difficult to solve. We decomposed it into two sub-optimization problems and approached the optimal solution using an alternating iteration algorithm. Finally, simulation results were used to validate the effectiveness of the proposed algorithm.

The contributions of our paper can be summarized as follows. The main innovation of this paper is to propose an innovative height measurement method for the meter-wave MIMO radar. This method, for the first time, combines beamforming technology with cognitive processing techniques for the height measurement of low-angle targets. Compared to the ML and GMUSIC algorithms, this method not only reduces complexity but is also applicable to multi-target scenarios. Furthermore, by introducing cognitive processing techniques, this approach provides a new perspective on the problem of the low-angle target height measurement.

**Notations:** Transpose and conjugate transpose are denoted by superscript ${\left(\right)}^{T}$ and ${\left(\right)}^{H}$, respectively. ⊗ denotes the Kronecker product. $I_{M}$ is the M×M dimensional identity matrix. vec(A) presents the vectorization of matrix A.

## 2. Signal Model

**Figure 3:**
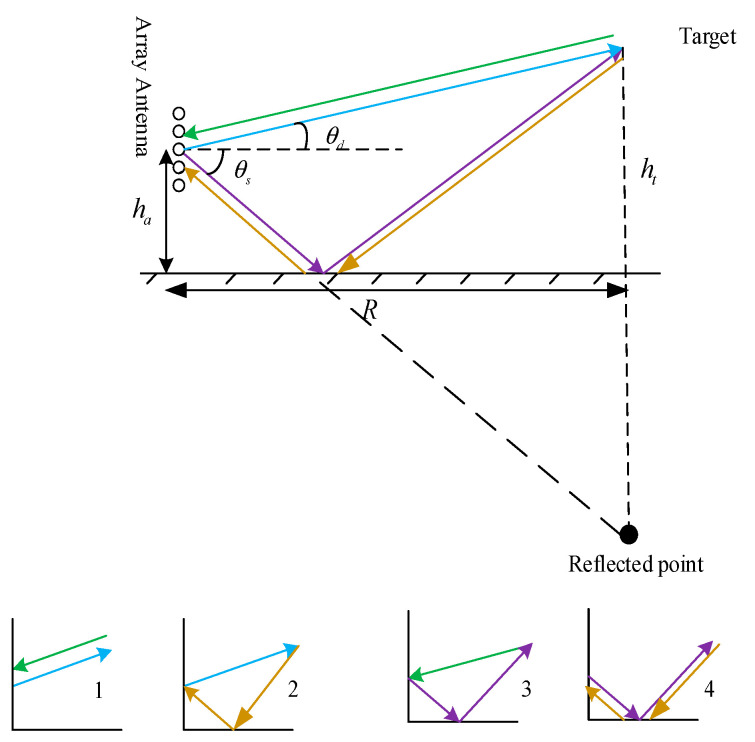
Signal model for meter-wave MIMO radar in low-elevation scenario.

This section establishes the signal model for the meter-wave MIMO radar based on the transmit weight matrix in a low-elevation scenario, as shown in Figure 3. The narrowband co-located meter-wave MIMO radar system consists of $M_{t}$ transmitted elements and $M_{r}$ receive elements, and the element spacing is half the wavelength d=0.5λ. It should be noted that for the target in the meter-wave MIMO radar, both transmitted and received multipath effects must be considered. Therefore, there are four transmission paths, respectively: the radar signal travels directly to the target and then directly back to the radar; the radar signal travels directly to the target and is reflected through the ground to the radar; the radar signal is reflected through the ground to the target and then travels directly back to the radar; and the radar signal is reflected through the ground to the target and then is reflected through the ground to the radar. $h_{a}$ is the height of the lowest array element and $h_{t}$ represents the height of the target. R denotes the projected distance between the radar and the target, while r denotes the range between the radar and the target.

Let $s(t)={\left[s_{1}(t),s_{2}(t),\ldots ,s_{M}(t)\right]}^{T}$ be the M×1 vector, assuming the waveforms are orthogonal. The transmit antennas emit linear combinations of these orthogonal waveforms. Therefore, the Mt×1 dimensional transmit signal vector can be expressed as follows:

(1) $$\varphi (t)={\left[x_{1}(t),\ldots ,x_{Mt}(t)\right]}^{T}=Wt*s(t)$$

where $Wt\in C^{Mt\times M}$ is the transmit weight matrix.

Then, the transmitted waveform signal of the array can be written as follows:

(2) $$x(t)={\left[a_{t}(\theta_{d})+\rho_{0}e^{-jk_{0}\Delta R}a_{t}(\theta_{s})\right]}^{T}\varphi (t)$$

where $\theta_{d}$ and $\theta_{s}$ are the angles of the direct and reflected paths, respectively. $\rho_{0}$ is the ground reflection coefficient, and $k_{0}=2\pi /\lambda$, ΔR represents the path difference between the direct wave and the reflected wave. $a_{t}(\theta_{d})$ and $a_{t}(\theta_{s})$ are the transmit array element guiding vectors, the values of which can be expressed as follows:

(3) $$\left\{\begin{array}{c} a_{t}(\theta_{d})\;={\left[e^{-j2\pi d_{1}\sin\theta_{d}/\lambda },\ldots ,e^{-j2\pi d_{Mt}\sin\theta_{d}/\lambda }\right]}^{T}\\ a_{t}(\theta_{s})\;={\left[e^{-j2\pi d_{1}\sin\theta_{s}/\lambda },\ldots ,e^{-j2\pi d_{Mt}\sin\theta_{s}/\lambda }\right]}^{T} \end{array}\right.$$

Thus, the received signal at the mr-th receive element is the following:

(4) $$\begin{array}{ll} z_{mr}(t,\tau )= & [a_{r,mr}(\theta_{d})+\rho_{0}e^{-jk_{0}\Delta R}a_{r,mr}(\theta_{s})]\beta (\tau )x(t)\;\\ & +\nu_{mr}(t,\tau ) \end{array}$$

where $\beta (\tau )=\alpha e^{j2\pi f_{d}\tau }$ is the multiple reflection coefficient of the target under different pulses, and $f_{d}$ is the Doppler frequency.

Then, the received signal of the entire array can be expressed as follows:

(5) $$\begin{array}{ll} z(t,\tau )= & \left[a_{r}(\theta_{d})+\rho_{0}e^{-jk_{0}\Delta R}a_{r}(\theta_{s})]\beta (\tau \right)\\ & *{\left[a_{t}(\theta_{d})+\rho_{0}e^{-jk_{0}\Delta R}a_{t}(\theta_{s})\right]}^{T}Wt*s(t)+\nu (t,\tau ) \end{array}$$

where

(6) $$\left\{\begin{array}{c} a_{r}(\theta_{d})\;={\left[e^{-j2\pi d_{1}\sin\theta_{d}/\lambda },\ldots ,e^{-j2\pi d_{Mr}\sin\theta_{d}/\lambda }\right]}^{T}\\ a_{r}(\theta_{s})\;={\left[e^{-j2\pi d_{1}\sin\theta_{s}/\lambda },\ldots ,e^{-j2\pi d_{Mr}\sin\theta_{s}/\lambda }\right]}^{T} \end{array}\right.$$

After performing matched filtering on the signal in Equation (5), we obtain the following:

(7) $$\begin{array}{c} Z=\int_{0}^{T_{p}}z(t,\tau )s{\left(t\right)}^{H}dt=[a_{r}(\theta_{d})+\rho_{0}e^{-jk_{0}\Delta R}a_{r}(\theta_{s})]\beta (\tau )\\ {}*{\left[a_{t}(\theta_{d})+\rho_{0}e^{-jk_{0}\Delta R}a_{t}(\theta_{s})\right]}^{T}Wt+V(\tau ) \end{array}$$

By vectorizing Equation (7), we obtain the following:

(8) $$\begin{array}{lll} Y & = & vec(Z)=[a_{r}(\theta_{d})+\rho_{0}e^{-jk_{0}\Delta R}a_{r}(\theta_{s})]\\ & & ⊗Wt^{H}[a_{t}(\theta_{d})+\rho_{0}e^{-jk_{0}\Delta R}a_{t}(\theta_{s})]\beta (\tau )+vec(V(\tau ))\\ & = & A\beta (\tau )+V \end{array}$$

where A is defined as the compound steering vector and its expression is the following:

(9) $$A=[a_{r}(\theta_{d})+\gamma a_{r}(\theta_{s})]⊗Wt^{H}[a_{t}(\theta_{d})+\gamma a_{t}(\theta_{s})]$$

where $\gamma =\rho_{0}e^{-jk_{0}\Delta R}$. V represents the noise after matched filtering and vectorization. Assuming that the original noise is Gaussian white noise, it follows from the literature [37] that after matched filtering and vectorization, the noise remains Gaussian white noise.

## 3. Analysis and Transformation of the Signal Model

The signal model for the meter-wave MIMO radar based on the transmit weight matrix under low-elevation scenarios is relatively complex. It needs to account not only for the multipath effects at both the transmitter and receiver but also for the coherence between the direct wave and reflected wave signals and the beamforming algorithm cannot directly handle coherent signals. To be compatible with beamforming algorithms, the signal model presented in the previous section requires further transformation. As described in Section 2, the signal model expression for the MIMO radar can be stated as follows:

(10) $$\begin{array}{ll} Y & =vec(Z)=[a_{r}(\theta_{d})+\gamma a_{r}(\theta_{s})]\;⊗Wt^{H}[a_{t}(\theta_{d})+\gamma a_{t}(\theta_{s})]\beta (\tau )+vec(V(\tau ))\\ & =A\beta (\tau )+V \end{array}$$

According to the signal model for the meter-wave MIMO radar under low-elevation scenarios, we can derive the following:

(11) $$\theta_{s}=-\arcsin(\sin\theta_{d}+2h_{a}R)\approx -\theta_{d}$$

According to the above equation and the expression for the steering vector, it is known that $a_{r}(\theta_{s})=a_{r}(-\theta_{d})$ and $a_{t}(\theta_{s})=a_{t}(-\theta_{d})$ hold true.

By observing the expression of the steering vector, we obtain the following:

(12) $$\left\{\begin{array}{c} a_{r}(\theta_{s})=a_{r}(-\theta_{d})=\kappa_{r}(\theta_{d})a_{r}(\theta_{d})\\ a_{t}(\theta_{s})=a_{t}(-\theta_{d})=\kappa_{t}(\theta_{d})a_{t}(\theta_{d}) \end{array}\right.$$

where

(13) $$\left\{\begin{array}{c} \kappa_{r}(\theta_{d})=diag[1,e^{j2k_{0}d\sin\theta_{d}}\ldots ,e^{j2k_{0}(Mr-1)d\sin\theta_{d}}]\\ \kappa_{t}(\theta_{d})=diag[1,e^{j2k_{0}d\sin\theta_{d}}\ldots ,e^{j2k_{0}(Mt-1)d\sin\theta_{d}}] \end{array}\right.$$

Therefore, the compound guiding vector A can be expressed as follows:

(14) $$\begin{array}{ll} A & =[a_{r}(\theta_{d})+\gamma a_{r}(\theta_{s})]⊗Wt^{H}[a_{t}(\theta_{d})+\gamma a_{t}(\theta_{s})]\\ & =[a_{r}(\theta_{d})+\gamma \kappa_{r}(\theta_{d})a_{r}(\theta_{d})]⊗Wt^{H}[a_{t}(\theta_{d})+\gamma \kappa_{t}(\theta_{d})a_{t}(\theta_{d})]\\ & =A_{r}(\theta_{d})⊗Wt^{H}A_{t}(\theta_{d}) \end{array}$$

(15) $$\left\{\begin{array}{c} A_{r}(\theta_{d})=[a_{r}(\theta_{d})+\gamma \kappa_{r}(\theta_{d})a_{r}(\theta_{d})]\\ A_{t}(\theta_{d})=[a_{t}(\theta_{d})+\gamma \kappa_{t}(\theta_{d})a_{t}(\theta_{d})] \end{array}\right.$$

Substituting Equation (14) into the signal model in Equation (10), we obtain the following:

(16) $$Y\;=A\beta (\tau )+V=A_{r}(\theta_{d})⊗Wt^{H}A_{t}(\theta_{d})\beta (\tau )+V$$

## 4. Height Measurement Algorithm for Meter-Wave MIMO Radar Based on the Joint Design of Transmitted Signals and Receive Filter

Based on the characteristics of beamforming technology to form nulls at interference locations, we treat the real target’s signal as an interference signal and assume the presence of a presumed target. Then, beamforming technology is utilized to process the received data to obtain the height of the low-elevation-angle target.

Assuming the direction of the presumed target is $\theta_{0}$, and the direction of the “interference signal” is $\theta_{d}$, the received signal of the array can be expressed as:

(17) $$y\;=A_{r}(\theta_{0})⊗Wt^{H}A_{t}(\theta_{0})\beta (\tau_{0})\;+A_{r}(\theta_{d})⊗Wt^{H}A_{t}(\theta_{d})\beta (\tau_{d})+V$$

In summary, our design problem is to estimate the elevation angle of the “interference signals” by jointly optimizing the transmit weight matrix Wt and the receive filter $w_{r}$, and then to obtain the target height.

Since a high SINR can improve the probability of target detection in the presence of interference, it is more advantageous for estimating the elevation angle and range of the “interference signals”. We aim to maximize the output SINR of the receiver by designing the transmitting weight matrix Wt and the receiving filter $w_{r}$. Therefore, the SINR can be written as:

(18) $$\mathrm{SINR}=\frac{\rho {\left|w_{r}^{H}A_{r}(\theta_{0})⊗Wt^{H}A_{t}(\theta_{0})\right|}^{2}}{w_{r}^{H}Rinw_{r}}$$

where

(19) $$\left\{\begin{array}{c} \rho =E[\beta (\tau_{0})\beta {\left(\tau_{0}\right)}^{H}]/\sigma_{v}^{2}=\sigma_{0}^{2}/\sigma_{v}^{2}\\ \eta =E[\beta (\tau_{d})\beta {\left(\tau_{d}\right)}^{H}]/\sigma_{v}^{2}=\sigma_{d}^{2}/\sigma_{v}^{2} \end{array}\right.$$

(20) $$\begin{array}{ll} Rin= & \eta (A_{r}(\theta_{d})⊗Wt^{H}A_{t}(\theta_{d})){\left(A_{r}(\theta_{d})⊗Wt^{H}A_{t}(\theta_{d})\right)}^{H}\\ & +I_{MtM} \end{array}$$

where $\sigma_{0}^{2}$ and $\sigma_{d}^{2}$ represent the transmitted power of the assumed target and the “interference signal”, respectively, and $\sigma_{v}^{2}$ represents the noise power.

Without loss of generality, we fixed the transmitting power to one, meaning that each antenna radiated energy 1/Mt at maximum power. Therefore, the problem can be described as follows:

(21) $$\Gamma :\left\{\begin{array}{c} \underset{Wt,w_{r}}{\max}\;\mathrm{SINR}\\ s.t.\;\sum_{m=1}^{M}{\left|W_{mt,m}\right|}^{2}=\frac{1}{Mt},\;mt=1,\ldots ,Mt \end{array}\right.$$

By observing Equation (21), we can find that the optimization problem Γ is non-convex and difficult to solve. Therefore, we converted it into two sub-optimization problems and approached the optimal solution through an alternating iteration algorithm. First, we optimized $w_{r}$ by fixing Wt, which is a classic Minimum Variance Distortionless Response (MVDR) problem. In this case, the receive filter can be expressed as follows:

(22) $$w_{r}=\frac{{\left(Rin\right)}^{-1}(A_{r}(\theta_{0})⊗Wt^{H}A_{t}(\theta_{0}))}{{\left(A_{r}(\theta_{0})⊗Wt^{H}A_{t}(\theta_{0})\right)}^{H}{\left(Rin\right)}^{-1}(A_{r}(\theta_{0})⊗Wt^{H}A_{t}(\theta_{0}))}$$

Secondly, with the receive filter $w_{r}$ fixed, we designed the transmit weight matrix Wt. This problem can be described as follows:

(23) Γ1:maxWt ρwrHf0vec(Wt)2ηwrHf1vec(Wt)2+wrHwr s.t. ∑m=1MWmt,m2=1Mt,mt=1,…,Mt

where $f_{i}=I_{K}⊗A_{r}{\left(\theta_{i}\right)}^{H}⊗A_{t}{\left(\theta_{i}\right)}^{H}$.

Considering that the aforementioned optimization problem $\Gamma_{2}$ is non-convex, we introduced the variable $\Psi =vec(Wt)vec{\left(Wt\right)}^{H}$ and reformulated the problem $\Gamma_{1}$ equivalently as follows:

(24) $$\Gamma_{1}^{'}:\left\{\begin{array}{c} \underset{\Psi }{\max}\;t\;\\ \begin{array}{c} s.t.\;\frac{\rho \mathrm{tr}(\Psi \Phi_{0})}{\eta \mathrm{tr}(\Psi \Phi_{1})+w_{r}^{H}w_{r}}\;\geq t\\ \mathrm{tr}(\Psi )=1,\Psi \geq 0,\mathrm{rank}(\Psi )=1 \end{array} \end{array}\right.$$

where $\Phi_{i}=f_{i}^{H}w_{r}w_{r}^{H}f_{i}$. The constraint  tr(Ψ)=1 indicates that the norms of the rows of $W_{t}$ are equal to 1/Mt, and the constraint Ψ≥0 indicates that a semi-definite constraint is present on Ψ, which is an affine constraint. Note that the rank constraint is not affine; therefore, we discarded it.

The above optimization problem is reformulated as follows:

(25) $$\Gamma_{1}^{\prime \prime }:\left\{\begin{array}{c} \underset{\Psi }{\max}\;t\\ \begin{array}{l} \;s.t.\;\rho \mathrm{tr}(\Psi \Phi_{0})-t(\eta \mathrm{tr}(\Psi \Phi_{1})+w_{r}^{H}w_{r})\geq 0\\ \mathrm{tr}(\Psi )=1,\Psi \geq 0 \end{array} \end{array}\right.$$

Notice that the optimization problem $\Gamma_{1}^{\prime \prime }$ is non-convex. However, if t is given, the feasible set is a convex set, meaning that the optimization problem $\Gamma_{1}^{\prime \prime }$ is a quasi-convex optimization problem. For a fixed t, the optimization problem $\Gamma_{1}^{\prime \prime }$ can be quickly solved using the CVX toolbox in MATLAB. To design $w_{t}=vec(Wt)$, we used randomization techniques to generate independent and identically distributed Gaussian vectors $w_{ti}\in C^{MtM\times 1}∼N(0,\Psi^{*}),i=1,2,\ldots ,N$, where N is the number of trials. To satisfy the energy constraint, we normalized these vectors.

(26) $$w_{ti}^{'}=\frac{w_{ti}}{\sqrt{w_{ti}^{H}w_{ti}}},i=1,\ldots ,N$$

From all $w_{ti}^{'}$, we selected the one $w_{t}$ that maximized the SINR as the optimal solution. The transmit weight matrix Wt could be obtained by performing the inverse operation of vectorization on $w_{t}$. Thus, we obtained the receive filter $w_{r}$ and the transmit weight matrix Wt. We then substituted Wt into Equation (22) to obtain the updated $w_{r}$, and continued to solve the optimization problem $\Gamma_{1}^{\prime \prime }$ to obtain the updated $w_{t}$. This process was repeated until the improvement in SINR was very small and could be neglected.

After obtaining the transmitting weight matrix Wt and receive filter $w_{r}$, beamforming was carried out by the following formula:

(27) $$B(\psi_{d})=w_{r}^{'}A(\psi_{d})$$

where $A(\psi_{d})=A_{r}(\psi_{d})⊗Wt^{H}A_{t}(\psi_{d})$.

To facilitate a comparison with other height measurement algorithms, the spectral peak was searched using the following formula:

(28) $$P(\psi_{d})=1/w_{r}^{'}A(\psi_{d})$$

When the search angle equals the angle of the ‘interference signal’, i.e., $\Psi_{d}=\theta_{d}$, the above formula forms a spectral peak at $\theta_{d}$. A summary of algorithm steps is shown in Algorithm 1.
**Algorithm 1.** Steps of algorithmInput: $\theta_{0}$, $\sigma_{0}^{2}$, $\theta_{d}$, $\sigma_{d}^{2}$, $Wt_{\left(0\right)}=\left(\sqrt{1/MM_{t}}\right)I_{M_{t}\times M}$

Output: $Wt^{*}$, $w_{r}^{*}$, $\theta_{d}$Set k=1

Step 1: Let $Wt_{\left(k\right)}=Wt_{\left(k-1\right)}$, and calculate $w_{r(k)}$ using Equation (22); Step 2: Calculate ${\mathrm{SINR}}_{\left(k\right)}$ using Equation (18); Step 3: Solve the problem $\Gamma_{1}^{\prime \prime }$ and obtain $Wt_{\left(k\right)}$;
  3.1Calculate $\Psi_{\left(k\right)}^{*}$ using Equation (25);  3.2Calculate $w_{ti(k)}^{'}$ using Equation (26), and calculate the corresponding ${\mathrm{SINR}}_{w_{ti(k)}^{'}}$ by satisfying Equation (23);  3.3Select $w_{ti(k)}^{'}$ that maximizes ${\mathrm{SIN}R}_{w_{ti(k)}^{'}}$, and which is noted as $\left(w_{ti(k)\max}^{'},{\mathrm{SINR}}_{w_{ti(k)\max}^{'}}\right)$;  3.4If ${\mathrm{SINR}}_{w_{ti(k)\max}^{'}}>{\mathrm{SINR}}_{\left(k\right)}$, let ${\mathrm{SINR}}_{\left(k\right)}{=\mathrm{SINR}}_{w_{ti(k)\max}^{'}}$, $w_{t(k)}=w_{ti(k)\max}^{'}$;  3.5$Wt_{\left(k\right)}=vec^{-1}(w_{t(k)})$;
Step 4: If ${(\mathrm{SINR}}_{\left(k\right)}{-\mathrm{SIN}R}_{0})/{\mathrm{SINR}}_{0}>\delta$, let ${\mathrm{SINR}}_{0}={\mathrm{SINR}}_{\left(k\right)}$, k=k+1, and return to step 1; otherwise, output $Wt^{*}=Wt_{\left(k\right)}$, $w_{r}^{*}=w_{r(k)}$; Step 5: Obtain the spectral peak results $P(\psi_{d})$ using Equation (28) and iterate this through $\psi_{d}$ and the angle corresponding to the maximum value of $P(\psi_{d})$ is the elevation angle estimation; Step 6: The height of the low-elevation target can be determined through the relationship between its elevation angle and height.

## 5. Algorithm Complexity Analysis

To more effectively evaluate the proposed algorithm’s performance, the computational complexity is analyzed in this section and compared with that of the GMUSIC algorithm and the ML algorithm. First, the complexity of both the GMUSIC algorithm and the ML algorithm mainly consists of three parts: the construction of the received data covariance matrix, the eigenvalue decomposition of the covariance matrix, and spectrum peak search. Therefore, the complexity of the GMUSIC algorithm can be expressed as $O[LMt^{2}Mr^{2}+Mt^{3}Mr^{3}+2\Theta Mt^{3}Mr^{3}]$, where L is the number of snapshots, Mt and Mr are the number of transmitted and received array elements, respectively, and Θ is the number of spectrum peak searches. Similarly, the complexity of the ML algorithm can be expressed as $O[LMt^{2}Mr^{2}+Mt^{3}Mr^{3}+\Theta (32MtMr+8Mt^{2}Mr^{2})]$.

Finally, the computational complexity of the proposed algorithm mainly comes from solving the optimization problem in Equation (25). Therefore, for Lt iterations, the total computational complexity is $O[LcLt{\left(MMt\right)}^{3.5}]$ [38], where Lc is the number of iterations needed to solve the optimization problem in Equation (25).

## 6. Simulation

This section validates the effectiveness and estimation performance of the algorithm through the simulation. The simulations were conducted on a personal computer equipped with an Intel(R) Core(TM) i7-1165G7 2.8 GHz CPU and 16 GB of memory by running Matlab R2018a code. In the simulation, we assumed that both the transmitted and received arrays were uniform linear arrays with element spacing equal to half a wavelength. Based on simulation experience, we balanced the optimization results of the algorithm and its computational complexity to set the parameters δ and N. We assumed that the initial radiation power of each element was equal. Therefore, we set δ=0.001, N=500, and $w_{t0}=vec\left({\left(\sqrt{1/M\times Mt}{Ι}_{Mt\times M}\right)}^{T}\right)$. The simulation conditions of the meter-wave MIMO radar are shown in Table 1. The Root Mean Square Error (RMSE) of the angle estimation is defined as follows:

(29) $$\mathrm{RMSE}(\theta )=\sqrt{\frac{1}{MC}\left({\sum_{i=1}^{MC}\left|{\hat{\theta }}_{i}-\theta \right|}^{2}\right)}$$

where MC denotes the number of Monte Carlo trials, ${\hat{\theta }}_{i}$ represents the estimated result of the i-th Monte Carlo trial for the incident angle, and θ is the true value of the target angle.

**Figure 4:**
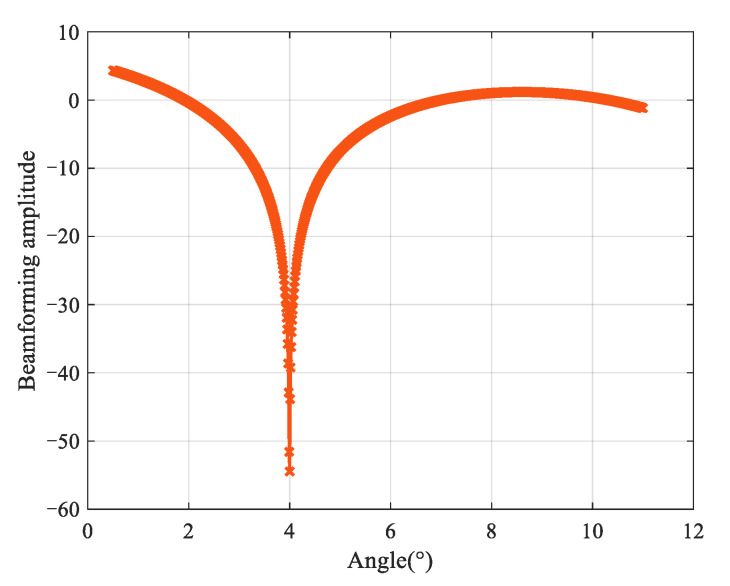
Beamforming result.

Experiment 1: Figure 4 shows the beamforming diagram of Equation (27). It is evident that Figure 4 forms null points in the direction of the interference signal, and the direction of arrival (DOA) of the low-elevation target can be obtained by identifying the positions of the null point. This demonstrates the effectiveness of the proposed algorithm. In the following simulations, to facilitate comparisons with the GMUSIC algorithm [39] and the ML algorithm [40], Equation (28) is used for the spectrum peak search.

**Figure 5:**
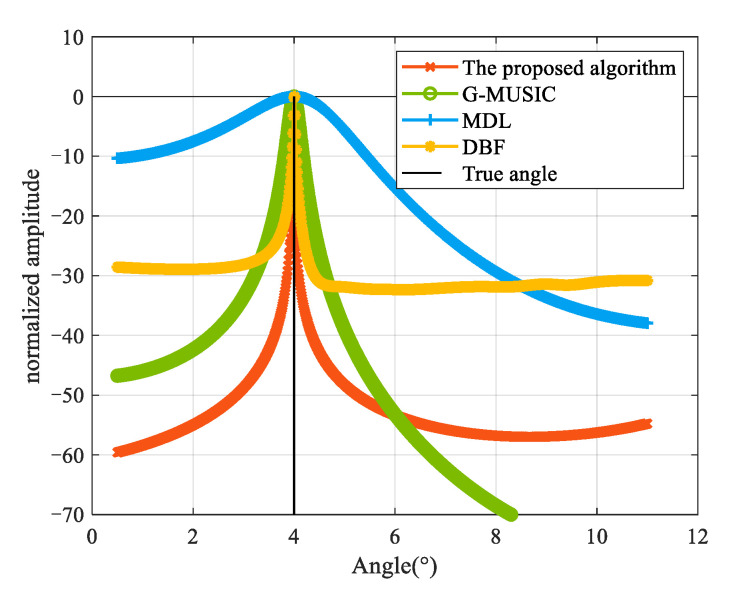
Spatial spectrum of the four algorithms.

Experiment 2: Figure 5 illustrates the spectral peak search results for the proposed algorithm and provides a comparison with the GMUSIC, ML, and DBF [21] algorithms. The true target angle is $\theta_{d}=4{}^{\circ}$, and the Signal-to-Noise ratio (SNR) is SNR=10 dB. From the figure, it can be seen that all four algorithms estimated the target elevation angle correctly. However, the proposed algorithm exhibited a sharper spectrum peak, making it easier to accurately identify the DOA of the low-elevation targets. This characteristic not only enhances the algorithm’s angular resolution but also demonstrates significant superiority in multi-target identification.

**Figure 6:**
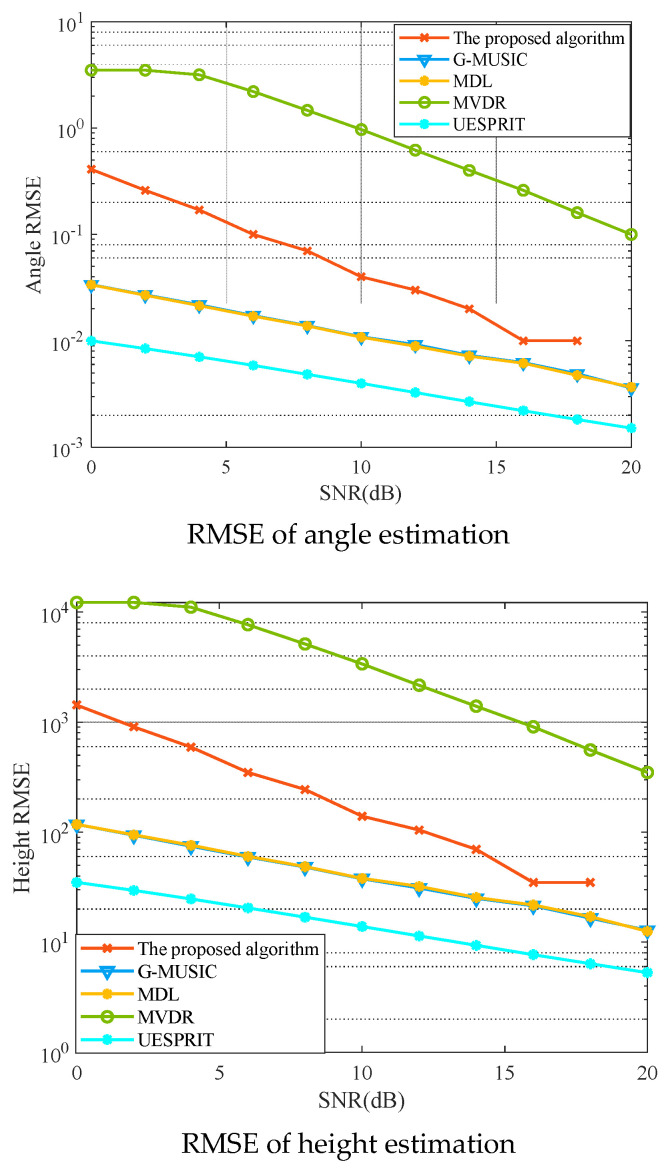
RMSE versus the SNR.

Experiment 3: Figure 6 shows the RMSE versus SNR of the proposed algorithm, the MVDR beamforming algorithm, the GMUSIC algorithm, the ML algorithm, and the UESPRIT algorithm [41]. From the figure, it can be seen that as the SNR increases, the RMSE for both angle estimation and height estimation decreases for all five algorithms. This is consistent with the theoretical analysis. In addition, compared with the MVDR beamforming algorithm, the performance of the proposed algorithm significantly improved. This improvement was achieved by adopting an iterative optimization approach for the transmit weight matrix and receive filter in contrast to the MVDR beamforming algorithm, which directly solves the receive filter based on the transmit weight matrix and is equivalent to performing only one iteration. Finally, with the increase in SNR, the proposed algorithm of the RMSE is closer to the GMUSIC algorithm, the ML algorithm, and the UESPRIT algorithm. At SNR=16 dB, the angle estimation error of the proposed algorithm is only 0.0038 degrees higher than that of the GMUSIC algorithm, and the corresponding height estimation error differs by 13 m. However, the proposed method is greatly reduced in computation complexity and has better real-time performance.

**Figure 7:**
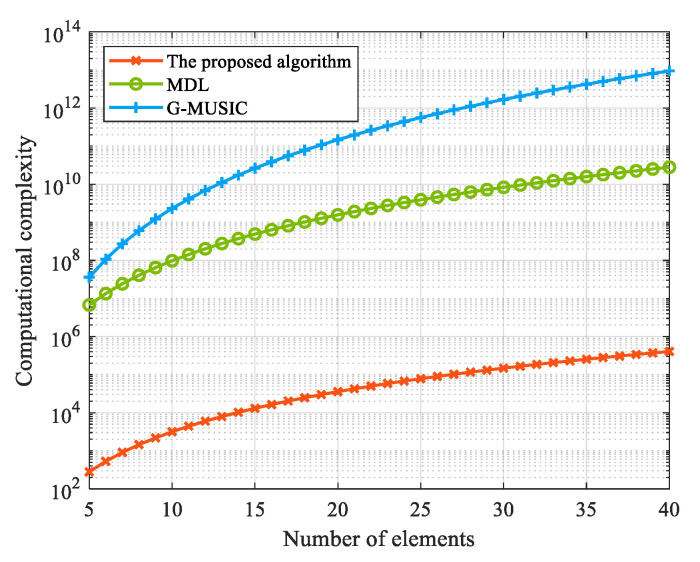
Computational complexity versus the number of array elements.

Experiment 4: Figure 7 shows the computational complexity curve of different algorithms with the number of elements. From the figure, it can be seen that the computational complexity of the proposed algorithm is significantly lower than that of the GMUSIC algorithm and the ML algorithm. Moreover, as the number of array elements increases, the proposed algorithm’s advantage in computational complexity becomes increasingly obvious.

**Figure 8:**
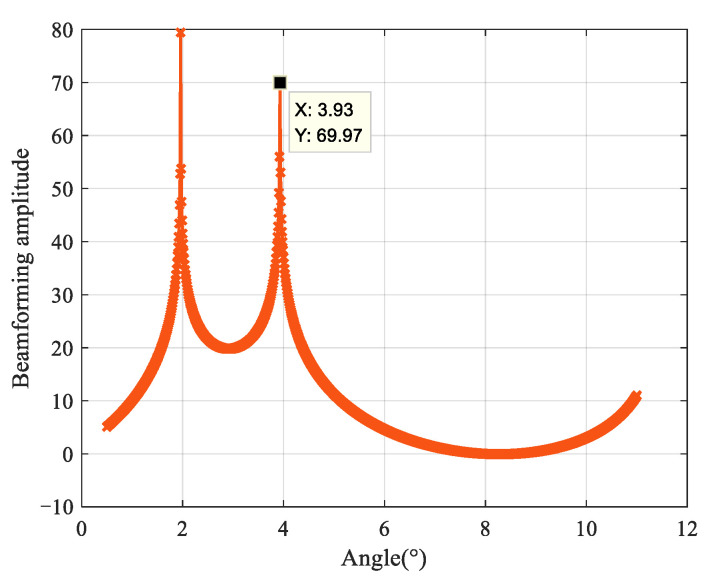
Angle estimation results for two targets simultaneously.

Experiment 5: Figure 8 analyzes the scenario of multiple targets in a multipath environment. According to the previous computational complexity analysis, in such situations, the number of unknown parameters in the compound steering vectors of the GMUSIC and ML algorithms increases, requiring multidimensional searches for angle estimation, which significantly increases the computational load. In contrast, the proposed algorithm uses an iterative search method, which can directly handle multiple targets, maintaining a relatively constant computational complexity.

Figure 8 shows the simultaneous estimation of the angles of two low-elevation targets using the proposed algorithm. In the simulation, the low elevation angles of target 1 and target 2 were 2° and 4°, respectively. From the figure, it can be seen that the proposed algorithm can effectively estimate the angles of two targets simultaneously. The RMSEs for the angle estimation of target 1 and target 2 are 0.04° and 0.07°, respectively. Based on the estimated elevation angles, the corresponding estimated heights of the targets are obtained. The maximum height offset for target 1 is approximately 139.54 m, and for target 2, it is approximately 243.76 m. These results meet the general design requirement of a height estimation error of 2% of the distance.

## 7. Conclusions

In this paper, a height measurement method based on the transmitted signal and receive filter design, combined with beamforming technology and the cognitive processing method, is proposed for the height measurement of the low-elevation region. The signal model for the meter-wave MIMO radar based on the transmit weight matrix is established under low-elevation scenarios. And the received data are processed to obtain the target’s elevation angle and height using a cognitive approach that jointly optimizes the transmitted signals and receive filter. In addition, the complexity of the algorithm is analyzed. The simulation results show that the proposed algorithm demonstrates superior performance in computational complexity and multi-target elevation estimation compared to the GMUSIC and ML algorithms.

## Figures and Tables

**Table 1:** Simulation conditions.

**Parameters**	**Symbols**	**Values**
Number of transmitted array elements	Mt	9
Number of received array elements	Mr	9
Reference carrier frequency	$f_{0}$	300 MHz
Angle of the presumed target	$\theta_{0}$	$9^{\circ }$
Angle of the “Interference Signal”	$\theta_{d}$	$4^{\circ }$
Reference antenna’s height	$h_{a}$	5 m
Range	r	200 km
Number of Monte Carlo trials	TrialAll	200
SNR threshold	δ	0.001
Number of randomized trials	N	500
Angle search range	angle-search	$0.5^{\circ }-11{}^{\circ}$
Angle search interval	interval	$0.01^{\circ }$

## Data Availability

The MATLAB codes can be obtained upon reasonable request by sending an email to qc337810137@163.com.
